# Impact of adenotonsillectomy on vocal emission in children^☆^^☆☆^

**DOI:** 10.1016/j.bjorl.2015.11.005

**Published:** 2015-12-14

**Authors:** Spyros Cardoso Dimatos, Luciano Rodrigues Neves, Jéssica Monique Beltrame, Renata Rangel Azevedo, Shirley Shizue Nagata Pignatari

**Affiliations:** a Department of Otolaryngology and Head and Neck Surgery, Universidade Federal de São Paulo (UNIFESP), São Paulo, SP, Brazil; b Human Communication Disorders, Department of Speech Therapy, Universidade Federal de São Paulo (UNIFESP), São Paulo, SP, Brazil; c Department of Speech Therapy, Universidade Federal de São Paulo (UNIFESP), São Paulo, SP, Brazil

**Keywords:** Voice, Tonsillectomy, Adenoidectomy, Pharyngeal tonsil, Palatine tonsil, Voz, Tonsilectomia, Adenoidectomia, Tonsila faríngea, Tonsila palatina

## Abstract

**Introduction:**

Adenotonsillectomy is the most common surgery performed by otolaryngologists in pediatric age, and one of the most frequently asked questions about the postoperative period is whether there is a potential for change in vocal pattern of these children.

**Objective:**

To evaluate the impact of adenotonsillectomy in the voice emission pattern of children with hypertrophy of palatine and pharyngeal tonsils.

**Methods:**

This is a prospective study in which we carried out perceptual auditory assessments and acoustic analysis of 26 children with adenotonsillar hypertrophy at three time points: before surgery, one month and three months after surgery. The following acoustic parameters were estimated using the Praat software: fundamental frequency, jitter, shimmer, and harmonic–noise ratio.

**Results:**

A statistically significant change was found between shimmer and harmonic–noise ratio during vowel /u/ production between the preoperative and 1st month postoperative time points. No significant differences were detected for acoustic parameters between preoperative analysis and that of the 3rd month post-operation.

**Conclusion:**

Transient changes in acoustic parameters occur in children with adenotonsillar hypertrophy submitted to adenotonsillectomy, progressing to normalization in the 3rd postoperative month.

## Resumo

**Introdução:**

Adenotonsilectomia é o procedimento cirúrgico mais realizado pelos otorrinolaringologistas em pacientes pediátricos, e entre as dúvidas mais frequentes a respeito do pós-operatório, inclui-se a possibilidade de modificações no padrão vocal dessas crianças.

**Objetivo:**

Avaliar o impacto da adenotonsilectomia no padrão de emissão vocal de crianças com hipertrofia de tonsilas palatinas e faríngea.

**Método:**

Trata-se de estudo prospectivo, em que foram realizadas a avaliação perceptiva-auditiva e a análise acústica da voz de 26 crianças com hipertrofia adenotonsilar em três oportunidades: no pré-operatório e no 1° e 3° meses após o procedimento cirúrgico. Os parâmetros acústicos frequência fundamental, jitter, shimmer e proporção harmônico-ruído foram avaliados por meio do programa Praat.

**Resultados:**

Houve uma alteração estatisticamente significante entre o shimmer e a proporção harmônico-ruído da emissão da vogal/u/entre o período pré-operatório e o 1° mês do pós-operatório. Não houve diferenças significantes dos parâmetros acústicos entre a análise pré-operatória e aquela realizada no 3° mês do pós-operatório.

**Conclusão:**

Crianças com hipertrofia adenotonsilar submetidas à adenotonsilectomia cursam com alterações transitórias dos parâmetros acústicos, evoluindo com a normalização dos mesmos no 3° mês do pós-operatório.

## Introduction

The voice is basically a product of three physiological processes: a constant expiratory airflow controlled by chest muscles; production of glottal sound through vibration of the vocal folds, and a change in this sound with amplification and muffling of sound frequencies resulting from the action of pharyngeal, oral and nasal resonant structures (vocal tract).^1^

Adenotonsillectomy is the most common surgery performed by otolaryngologists, especially in children. Among the most frequently voiced concerns regarding this procedure are questions about changes in vocal patterns after surgery and whether they are temporary or permanent.

According to Mora et al., hypertrophic palatine tonsils reduce the oropharyngeal air space and push the tongue forward, causing mouth breathing, abnormal nasality and a muffled voice.^2^ It is also reported that adenoid and tonsil hypertrophy cause obstruction of the nasopharyngeal region and a decreased mobility of velopharyngeal muscles (*i.e.* soft palate).^2^

Although it is the most studied, nasality is not the only form of voice alteration that can occur after adenotonsillectomy. With vocal tract modification, there can be changes in voice quality due to phonation instability, as a consequence of changes in the vibration pattern of the vocal folds.^2^, ^3^

However, to date, few studies have assessed vocal emission after adenotonsillectomy, and most of these did so using only subjective measures (perceptual-auditory voice analysis).

The aim of this study is to assess the impact of adenotonsillectomy on the pattern of vocal emission of children with hypertrophy of palatine and pharyngeal tonsils.

## Methods

This is a prospective study with surgical intervention and postoperative monitoring, which began in January 2009 and ended in December of the same year. Twenty-six children, between 5 and 10 years of age, suffering from palatine and pharyngeal tonsil hypertrophy and with prior indication for adenotonsillectomy, were monitored.

This study was approved by CEP, according to Resolution 196/96 of the National Ethics in Research Committee – CONEP, dealing with guidelines and regulatory standards for research involving human subjects.

Our children were submitted to otorhinological examination, which consisted in a detailed history, physical examination, and transnasal fiberoptic laryngoscopy.

The inclusion criteria for this study were: palatine tonsil hypertrophy grades III or IV, according to the scale proposed by Brodsky,^4^ pharyngeal tonsil hypertrophy with >70% obstruction of the nasopharynx diagnosed by direct visualization with the use of nasal fiberoptic endoscopy and due consent of the legal representative for the child's participation in the study, according to the proposed method, and after a detailed study explanation and the Free and Informed Consent Term (FICT) signature.

Patients with dysphonia or changes in vocal emission, upper or lower airway infection, previously submitted to speech therapy, with craniofacial malformation, or with neurological syndromes were excluded.

All children underwent adenotonsillectomy carried out by the same medical team, always under the supervision of the researcher. The surgical technique consisted of cold steel adenotonsillectomy, and hemostasis was carried out with simple sutures with absorbable suture (Catgut 2.0).

The vocal emission recordings were performed in a quiet environment with the use of a Samson C03 professional microphone (Samson Technologies, USA) at a distance of approximately 5 cm from the child's mouth; the material obtained was edited in Sound Forge 8.0 software (Sony, Japan), from which emission parts of the sustained vowels /a/, /i/ and /u/ and parts of chained speech (automatic sequence and spontaneous speech) were extracted.

The vocal emissions of these children were recorded in three circumstances, namely:

• 1st Time point (T0) – preoperative period;
• 2nd Time point (T30) – 1 month after the surgery;
• 3rd Time point (T90) – 3 months after the surgery.

The perceptual-auditory evaluation of voice quality (spontaneous speech and automatic sequence) was performed by three experienced speech therapists (blinded for this study), and the visual analog scale (VAS), validated by Yamasaki et al.,^5^ was used as the evaluator parameter.

The VAS corresponds to a 100 mm-long line in which the judge is encouraged to mark a point that represents his/her experienced feeling at the moment in regards to the voice recording presented. On VAS, each millimeter corresponds to one degree of deviation, where the extreme left represents absence of vocal change, and the extreme right is the maximum degree of change. The cutoff value to discriminate between a normal and altered voice is 35.5 mm.^5^

A computerized acoustic analysis of the child's voice was carried out for evaluation of vowels /a/, /i/, and /u/, with the use of the Praat software (Phonetic Sciences, University of Amsterdam, Netherlands).^6^

The acoustic parameters analyzed were the following:

• Fundamental frequency (Fo) – The number of glottic cycles per second, reflecting the biomechanical characteristics of the vocal folds and their relation with subglottic pressure;
• Jitter (J) – Cycle-to-cycle variations of fundamental frequency;
• Shimmer (S) – A measure that quantifies cycle-to-cycle fluctuations in the intensity of the glottal excitation; and
• Harmonic–noise ratio (HNR) – An established relationship between the sounds produced by the larynx *versus* noises that interfere with voice production.

A statistical analysis comparing preoperative and postoperative data was conducted, and the significance level was set at 5% (*p* < 0.05). For this purpose, the following tests were applied:

Friedman test for each variable, in order to verify possible differences among the three observation time points.

Wilcoxon test using Bonferroni correction, in order to observe in what the observational time points chosen differ from the others, when in a pairwise comparison.

## Results

Twenty-six children aged between 5 and 10 years (mean = 7.15 years) (16 males and 10 females) were evaluated.

### Perceptive auditory voice evaluation

For the perceptual auditory analysis carried out using a VAS, a statistically significant difference among the three time points was found (Table 1).

**Table 1:** Perceptual-auditory assessment (VAS).

Perceptual-auditory assessment	*n*	Mean	Standard deviation	Minimum	Maximum	Percentile 25	Median	Percentile 75	Significance (*p*)
T0	26	1.442	1.087	0.200	3.900	0.500	0.850	2.225	0.002
T30	26	1.988	1.052	0.300	4.000	1.000	2.100	2.925	
T90	26	1.473	0.981	0.200	3.200	0.700	1.200	2.500	

By applying the Wilcoxon test using Bonferroni correction, we found a statistically significant difference between the assessment carried out at the 1st month after the surgery and that undertaken in the 3rd month postoperatively (*p* = 0.005) and a trend showing that the time points before the surgery and at 1 month postoperatively are statistically different (Table 2).

**Table 2:** Perceptual-auditory assessment analysis.

Pair of variables analyzed	Significance (*p*)
T30 – T0	0.039
T90 – T0	0.903
T90 – T30	0.005^a^

a Bonferroni's alpha = 0.016667.

### Computerized acoustic voice analysis

#### Vowel /a/

Regarding the vowel /a/, no statistically significant difference was found among the studied parameters (fundamental frequency, jitter, shimmer, and HNR) in the three observational time points (Table 3, Table 4, Table 5, Table 6).

**Table 3:** Fundamental frequency – Vowel /a/.

Fundamental frequency	*n*	Mean	Standard deviation	Minimum	Maximum	Percentile 25	Median	Percentile 75	Significance (*p*)
T0	26	238.403	30.873	201.259	310.992	215.525	225.529	256.087	0.382
T30	26	240.826	31.719	190.438	324.569	219.385	237.142	252.817	
T90	26	232.375	36.962	137.915	305.835	211.768	230.933	257.945

**Table 4:** Jitter – Vowel /a/.

Jitter	*n*	Mean	Standard deviation	Minimum	Maximum	Percentile 25	Median	Percentile 75	Significance (*p*)
T0	26	0.887	1.017	0.192	4.155	0.352	0.526	0.899	0.707
T30	26	0.698	0.358	0.205	1.899	0.470	0.628	0.834	
T90	26	0.595	0.340	0.239	1.493	0.391	0.467	0.685

**Table 5:** Shimmer – Vowel /a/.

Shimmer	*n*	Mean	Standard deviation	Minimum	Maximum	Percentile 25	Median	Percentile 75	Significance (*p*)
T0	26	8.621	4.564	2.521	20.449	4.482	8.451	10.816	0.607
T30	26	5.893	2.608	1.254	11.903	3.880	5.766	7.617	
T90	26	6.965	3.674	2.618	16.795	4.392	6.026	7.425

**Table 6:** Harmonic–noise ratio – Vowel /a/.

Harmonic–noise ratio	*n*	Mean	Standard deviation	Minimum	Maximum	Percentile 25	Median	Percentile 75	Significance (*p*)
T0	26	14.098	4.367	4.234	20.818	12.821	14.289	17.018	0.857
T30	26	14.966	3.619	7.393	22.320	12.377	14.876	17.256	
T90	26	14.940	3.179	8.043	22.237	13.572	14.875	17.358	

#### Vowel /i/

The analysis of the emission of sustained vowel /i/ at the three observational time points showed differences in the fundamental frequency of the voice between the preoperative period and 3 months postoperatively (Table 7, Table 8).

**Table 7:** Fundamental frequency – Vowel /i/.

Fundamental frequency	*n*	Mean	Standard deviation	Minimum	Maximum	Percentile 25	Median	Percentile 75	Significance (*p*)
T0	26	260.239	40.543	217.994	375.138	231.627	250.910	272.441	0.048^a^
T30	26	256.229	38.329	190.388	353.938	227.330	250.870	277.795	
T90	26	245.710	34.407	192.366	316.837	219.197	239.116	274.424	

a Bonferroni's alpha = 0.016667.

**Table 8:** Fundamental frequency analysis (Fo) – Vowel /i/.

Pair of variables	Significance (*p*)
T30 – T0	0.501
T90 – T0	0.009
T90 – T30	0.058

Statistically significant changes were not identified in the remaining parameters (jitter, shimmer, HNR) (Table 9, Table 10, Table 11).

**Table 9:** Jiitter – Vowel /i/.

Jitter	*n*	Mean	Standard deviation	Minimum	Maximum	Percentile 25	Median	Percentile 75	Significance (*p*)
T0	26	0.907	1.066	0.190	5.585	0.310	0.618	1.007	0.240
T30	26	0.478	0.228	0.197	1.239	0.341	0.456	0.515	
T90	26	0.507	0.353	0.142	1.968	0.310	0.450	0.542

**Table 10:** Shimmer – Vowel /i/.

Shimmer	*n*	Mean	Standard deviation	Minimum	Maximum	Percentile 25	Median	Percentile 75	Significance (*p*)
T0	26	7.033	4.625	2.479	20.331	3.181	6.146	8.910	0.089
T30	26	4.151	2.399	0.651	12.832	2.931	3.694	4.729	
T90	26	4.864	3.424	1.551	19.216	3.151	3.788	5.876

**Table 11:** Harmonic–noise ratio – Vowel /i/.

Harmonic–noise ratio	*n*	Mean	Standard deviation	Minimum	Maximum	Percentile 25	Median	Percentile 75	Significance (*p*)
T0	26	18.693	4.769	4.601	27.854	15.402	18.436	22.023	0.341
T30	26	20.755	2.712	15.465	25.920	19.308	21.325	22.186	
T90	26	20.575	3.870	8.925	28.471	18.303	20.193	23.446	

#### Vowel /u/

Regarding the vowel /u/, no statistically significant difference was detected in fundamental frequency and jitter (Table 12, Table 13).

**Table 12:** Fundamental frequency – Vowel /u/.

Fundamental frequency	*n*	Mean	Standard deviation	Minimum	Maximum	Percentile 25	Median	Percentile 75	Significance (*p*)
T0	26	261.170	44.299	174.045	347.238	229.347	252.526	290.321	0.764
T30	26	264.623	50.040	204.910	426.145	223.374	257.706	283.011	
T90	26	256.735	38.025	207.698	327.897	222.196	247.372	284.049

**Table 13:** Jitter – Vowel /u/.

Jitter	*n*	Mean	Standard deviation	Minimum	Maximum	Percentile 25	Median	Percentile 75	Significance (*p*)
T0	26	1.134	1.435	0.227	6.867	0.388	0.724	1.025	0.076
T30	26	0.531	0.225	0.273	1.007	0.371	0.458	0.615	
T90	26	0.539	0.215	0.245	1.243	0.380	0.527	0.658	

A statistically significant difference was detected for shimmer and HNR measures between the preoperative recording and that undertaken at the 1st month after the surgery (Table 14, Table 15, Table 16, Table 17).

**Table 14:** Shimmer – Vowel /u/.

Shimmer	*n*	Mean	Standard deviation	Minimum	Maximum	Percentile 25	Median	Percentile 75	Significance (*p*)
T0	26	8.117	5.282	2.908	22.404	4.448	5.968	9.990	0.019*
T30	26	4.557	2.302	0.808	11.614	3.251	4.138	5.088	
T90	26	6.296	4.179	2.126	19.265	3.474	4.829	7.926	

* *p* < 0.05.

**Table 15:** Harmonic–noise ratio – Vowel /u/.

Harmonic–noise ratio	*n*	Mean	Standard deviation	Minimum	Maximum	Percentile 25	Median	Percentile 75	Significance (*p*)
T0	26	19.175	6.271	4.529	27.033	16.010	21.208	24.214	0.004*
T30	26	23.761	3.230	15.513	30.059	22.222	24.158	26.103	
T90	26	22.116	4.090	10.796	28.865	20.470	22.847	25.045	

* *p* < 0.05.

**Table 16:** Analysis of shimmer (S) – Vowel /u/.

Pair of variables	Significance (*p*)
T30 – T0	0.003^a^
T90 – T0	0.159
T90 – T30	0.058

a Bonferroni's alpha = 0.016667.

**Table 17:** Harmonic–noise ratio (HNR) – Vowel /u/.

Pair of variables	Significance (*p*)
T30 – T0	0.004^a^
T90 – T0	0.069
T90 – T30	0.026

a Bonferroni's alpha = 0.016667.

## Discussion

The vocal tract can be defined as the set of anatomical structures located above the glottis that modifies the sounds produced by vibrations of the vocal folds, because of resonance effects on those sounds due to physical attributes of the tract.^7^

This resonance is the product of a three-dimensional configuration of the vocal tract, the tone of its walls, characteristics of the mucosal lining and its viscoelastic properties.^8^

Any change in these features will impact the sound propagation and the definition of vocal formants. The frequencies that define the first formant, for instance, may vary according to changes in jaw positioning, while the second formant is influenced by the tongue position.^7^ The third formant relates to resonance of the region above the vocal folds; this area consists of the laryngeal ventricles, aryepiglottic folds and vestibular folds, while the fourth and fifth formants are more dependent on the vocal tract length than on the articulators’ position.^7^

It is assumed that changes in vocal tract anatomy resulting from surgical procedures may modify the subject's vocal characteristics.

Mora et al. related that the presence of hypertrophic tonsils reduces the oropharyngeal space, project the tongue forward and cause hypernasality, mouth breathing and muffled voice. In their study, 40 children with ages ranging from 4 to 14 years were submitted to an acoustic analysis before and 30 days after adenotonsillectomy. After the surgery, these authors observed a statistically significant improvement in all parameters: fundamental frequency, jitter, shimmer, and HNR, as well as others.^2^

Considering that the adenoid tissue is a space-consuming structure, Salami et al. proposed that its removal would result in changes in the nasopharyngeal anatomy. Similarly, enlarged tonsils and adenoids can block the nasopharyngeal airflow and influence soft palate mobility. After acoustically analyzing children in the preoperative period and 1 month after adenotonsillectomy, these authors found improvement in voice quality and in the entire group of acoustic parameters analyzed.^3^

Jarboe et al. studied the impact of adenotonsillectomy in professional singers. These authors retrospectively evaluated through telephone questionnaires 23 patients in a late postoperative period and found that the vast majority showed improvement in vocal quality after surgery; in only five patients did they identify a loss of vocal quality in the early postoperative period (1–4 months),and all experienced recovery. Despite the results of their study, Jarboe et al. point out that, an improvement in voice quality does not *per se* justify an indication of adenotonsillectomy.^9^

On the other hand, Chuma et al., in a prospective study of 23 children who underwent acoustic analysis before and 3 months after adenotonsillectomy, concluded that the removal of tissue from the oropharynx exerts minimal quantitative and qualitative (perceptual) impact on several aspects of vocal function.^10^

Subramanian et al. evaluated the effects of tonsillectomy with or without adenoidectomy through an acoustic analysis of 20 patients. These authors recorded the vocal register the day before the procedure and at 1 month postoperatively, and found decreases in shimmer. However, their patients did not undergo a long-term acoustic analysis; thus, this finding may correspond to a transient post-surgical change. Unlike the present study, the authors also evaluated the nasality and observed that this factor diminished after the surgery.^11^

Kara et al. conducted an analysis of the effects of surgical removal of pharyngeal tonsils on their patients’ voice and speech. These authors evaluated 36 children with pharyngeal tonsil hypertrophy before and 3 months after surgery, and found a significant change in nasality and also in the 3rd and 4th formants. In line with our study, no change in fundamental frequency, shimmer and HNR was found. The authors state that adenoidectomy procedures may affect vocal resonance and nasality, by modifying the shape and size of the nasopharynx and upper respiratory tract.^12^

To Lundeborg et al., adenotonsillar hypertrophy affects the voice quality, both in perceptive and acoustic analyses. These authors studied vocal outcomes in 67 children after adenotonsillectomy and concluded that those patients with tonsil and adenoid hypertrophy preoperatively exhibited hyponasality, a deeper pitch and higher perturbation measures, compared to the control group and to a postoperative evaluation. However, there was no significant change in fundamental frequency before and after surgery, thus demonstrating vocal tract interference in the psychoacoustic sense of frequency.^13^

In our study, the group of patients studied consisted of children with hypertrophy of the tonsils and adenoids, aged between 5 and 10 years old. It was decided to exclude children under 5 years because they could face difficulties executing the required tasks, since their oral code is not yet fully established. Children older than 10 years were not included in our study due to the proximity to their voice change, a fact that could interfere with the analysis of the collected results.

For a proper investigation of the vocal pattern of these children, two tests were chosen, namely: perceptive auditory and acoustic voice analysis.

The perceptive auditory voice analysis allows an assessment of vocal perception for two vocal aspects: glottal source and resonant filter.^7^ If carried out during a chained speech (counting numbers, narrating months of the year, or reading a predefined text), this analysis is more comprehensive and also includes vocal aspects related to articulation and resonance, thus being considered by many authors as the gold standard of vocal assessment.^7^ This kind of analysis allows the characterization of vocal quality and the quantification of phonation deviation in the face of a given stimulus. Being an essentially acoustic phenomenon of subjective nature, its utilization requires previous training and experience of those professionals in charge of the evaluation.^7^

We found a statistically significant difference between the assessments in the 1st and 3rd months after the surgery; we also noticed a tendency in favor of a difference between time points before and at the 1st month after the surgery. These findings support the hypothesis that adenotonsillectomy procedures are responsible for transient changes in the pattern of vocal emission, creating a temporary phonation instability, that disappears throughout the postoperative period.

The acoustic analysis of human voice raises greater interest, by involving objective, observer-judge-independent measures. According to Vieira et al., these measures can help in therapeutic monitoring, contributing directly by verifying the effectiveness of a particular strategy or proposed therapeutic approach. Vieira et al. also state that acoustic registers and measurements can bolster the defense of the physician or speech therapist in legal disputes where the effectiveness of a vocal treatment outcome is being questioned.^8^

In this study, the vowels /a/, /i/ and /u/ were chosen to carry out the computerized acoustic analysis, and data on fundamental frequency, jitter, shimmer, and HNR were collected.

These three vowels were chosen because they form the vertices of the polygon representing the average of the frequencies of the first two formants of oral vowels of the Brazilian Portuguese language, as defined by Behlau et al.^7^

The vowel /a/ is an oral, central, low, and open vowel; the vowel /i/ is an oral, anterior, high, closed, not-rounded vowel; and the characteristics of vowel /u/ are those of an oral, posterior, high, closed, and rounded vowel.

In the analysis of the data produced, we found statistical differences only in relation to shimmer and HNR for vowel /u/ between the preoperative evaluation and that carried out at 1 month after surgery. However, this modification may be transitory, since no statistical difference between the preoperative evaluation and that performed three months after surgery was found.

By being a posterior vowel, /u/ proved to be most affected by modifications of the anatomy stemmed from adenoid/tonsillar tissue removal at the end of the first month of the postoperative period – a fact compensated later, in our longer postoperative time point.

Likewise, the only statistically significant difference found in the sustained emission of vowel /i/ was due to the observation of the fundamental frequency between the preoperative assessment and that carried out three months after the surgery.

The results presented in this paper demonstrate a partial agreement with the results of other studies that evaluated the effects of adenotonsillectomy at one month postoperatively; these studies found significant differences in all parameters (fundamental frequency, jitter, shimmer and HNR).^2^, ^3^ Perhaps some methodological and sample standardization variations may justify this variation of data. However, these studies did not carry out a medium-term (time point = 3 months) assessment of the children.

The results of this study provide data that suggest that children with enlarged tonsils and adenoids submitted to adenotonsillectomy exhibit transient changes in their vocal quality/acoustic parameters, but experience the return to preoperative levels 90 days after surgery.

## Conclusion

Children with adenotonsillar hypertrophy submitted to adenotonsillectomy are affected by transient changes of their acoustic parameters, progressing to normalization in the 3rd month postoperatively.

## Conflicts of interest

The authors declare no conflicts of interest.
